# Rapid Growth and Persistent Geographic Concentration of Recorded Outpatient Corneal Cross-Linking Production in Brazil’s Unified Health System, 2017-2025

**DOI:** 10.7759/cureus.114728

**Published:** 2026-08-18

**Authors:** Matheus G Maués, Victória Amin, Manuela Rezende, Luis Rodrigues

**Affiliations:** 1 Medicine, Centro Universitário do Pará, Belém, BRA

**Keywords:** corneal cross-linking (cxl), geographic concentration, health services, keratoconus (kc), outpatient production, unified health system

## Abstract

Background

Corneal cross-linking (CXL) is the principal intervention used to stabilize progressive keratoconus. The procedure was incorporated into Brazil's Unified Health System (Sistema Único de Saúde (SUS)) in 2016 and became operational in national administrative systems in 2017. The geographic distribution of outpatient CXL production within the SUS has not been fully characterized.

Objective

To examine temporal growth, geographic distribution across federative units, geographic concentration, and the provider legal categories associated with recorded approved outpatient CXL production in the SUS from 2017 through 2025.

Methods

This nationwide ecological time-series study used public, aggregated data from the SUS Outpatient Information System (SIA/SUS). Approved quantities for procedure 04.05.05.040-2 were analyzed by processing year, federative unit, macroregion, and provider legal category. Indicators included annual volume, the number of active federative units, state and regional shares, the Herfindahl-Hirschman Index (HHI), effective number of federative units, Gini coefficient, and provider-category composition. Because 2017 represented a partial implementation year, long-term growth was assessed from 2018 to 2025. Analyses were descriptive and did not use inferential tests because the dataset covered the complete national aggregated series available for the procedure.

Results

A total of 12,216 approved outpatient procedures were recorded from 2017 to 2025. Annual production increased from 263 procedures in 2018 to 3,415 in 2025, a 12.98-fold increase corresponding to a compound annual growth rate of 44.2%. The number of federative units recording at least one procedure increased from five to 16. Despite this nominal diffusion, São Paulo accounted for 69.1% of national production in 2025 and the Southeast for 77.7%. The HHI across federative units was 0.497, equivalent to only 2.01 equally productive units, and the Gini coefficient was 0.893. Nine federative units recorded no outpatient production in any year. The North accounted for 0.7% of procedures in 2025. Establishments outside public administration accounted for 89.1% of that year's recorded production.

Conclusions

Recorded approved outpatient CXL production in the SUS increased rapidly and was observed in a growing number of federative units, but procedure volume remained heavily concentrated in a small number of states. These findings describe the geographic distribution of recorded outpatient production rather than individual access, service availability, or population need. A more complete assessment will require age-specific population denominators, patient residence, interstate referral flows, hospital records, and establishment-level data.

## Introduction

Keratoconus is a progressive corneal ectasia characterized by stromal thinning, biomechanical weakening, and irregular corneal protrusion. The disease typically becomes apparent during adolescence or early adulthood and may lead to irregular astigmatism, substantial visual impairment, dependence on specialty contact lenses, corneal scarring, and, in advanced cases, keratoplasty. Current clinical guidance, including the 2026 international consensus published after the study period, emphasizes early recognition of progression and timely intervention as central elements of contemporary keratoconus care [1]. A 2025 systematic review and meta-analysis estimated a pooled global prevalence of 289.1 cases per 100,000 population, representing more than 23.7 million affected individuals, although estimates varied markedly across geographic settings and study methods [2].

Corneal cross-linking (CXL) combines riboflavin with ultraviolet-A irradiation to increase stromal biomechanical stiffness and slow or halt ectatic progression [3]. Randomized trials and long-term follow-up studies support its effectiveness in stabilizing progressive keratoconus [4-6]. At the population level, the introduction of CXL has been associated with fewer keratoplasties performed for keratoconus [7,8]. From the perspective of the Sistema Único de Saúde (SUS), a recent economic evaluation found CXL to be highly cost-effective and potentially capable of preventing corneal transplantation over a patient's lifetime [9].

In Brazil, CXL was incorporated into the SUS through Ordinance No. 30 of September 20, 2016 [10,11]. The national clinical-use protocol was established by Ordinance No. 486 of March 6, 2017, which created procedure code 04.05.05.040-2, defined eligibility between 15 and 45 years of age and ICD-10 code H18.6, and permitted recording either as an individualized outpatient procedure or as the principal procedure on a hospital authorization [12]. The ordinance also assigned state, Federal District, and municipal health authorities responsibility for organizing the care network, designating referral services, and establishing patient pathways.

Formal incorporation of a technology and evidence of clinical effectiveness do not establish how recorded production is geographically distributed within a large health system. Administrative production data can therefore provide a complementary health-services perspective by identifying where procedures are recorded, how recorded volumes change over time, and whether national growth is geographically concentrated. Local patterns of recorded production may be influenced by equipment, professional training, corneal tomography, referral network organization, contracting arrangements, and providers' administrative capacity to submit claims. Research on health-technology implementation in the SUS indicates that national guidance often specifies incorporation and financing more clearly than local operational pathways [13]. At the same time, regional disparities persist in the use of specialized services and in the distribution of health-care infrastructure across Brazil [14,15].

National totals may therefore conceal substantial geographic heterogeneity. An increase in the number of federative units with any recorded activity does not necessarily indicate meaningful decentralization: a unit may be classified as active after only a few procedures while most national production remains concentrated in a single state. The Herfindahl-Hirschman Index (HHI), the effective number of federative units, and the Gini coefficient can distinguish nominal diffusion from the actual distribution of procedure volume.

To our knowledge, recorded outpatient CXL production in the SUS has not previously been examined nationwide simultaneously in terms of temporal growth, geographic distribution across federative units, concentration of production, and provider legal category. This study evaluated approved outpatient CXL production recorded in the SUS Outpatient Information System (SIA/SUS) from 2017 through 2025, characterized its regional and state-level geographic distribution according to place of care, and assessed whether national growth in recorded production was accompanied by reduced geographic concentration.

## Materials and methods

Study design and reporting framework

We conducted a nationwide ecological time-series study using Brazil’s 27 federative units-26 states and the Federal District-as the geographic units of analysis. The study period extended from January 2017 to December 2025. The analysis characterizes recorded approved outpatient production and its geographic distribution according to place of care; it does not directly measure patient access, equity, service availability, population coverage, or individual utilization. Reporting was informed by applicable principles of the Strengthening the Reporting of Observational Studies in Epidemiology (STROBE) statement [16], recognizing the ecological time-series design of the present study.

Data source

Data were obtained from the TabNet interface of the SUS Outpatient Information System (SIA/SUS), using the “place of care” option. Place of care identifies the location of the establishment that submitted the procedure rather than the patient's place of residence. Accordingly, all geographic analyses in this study refer to the location of recorded outpatient production and should not be interpreted as the geographic distribution of patients receiving CXL. The temporal variable was processing year, and the selected measure was approved quantity, defined as the volume accepted by the information system for reimbursement purposes [17].

Two independent tabulations of the same procedure were used: approved quantity by federative unit and approved quantity by provider legal category, each extracted separately for every year from 2017 through 2025. National totals from the two tabulations were compared year by year as an internal consistency check. No discrepancies were identified.

Procedure and scope of care

The analysis focused on procedure 04.05.05.040-2, “Radiation for corneal cross-linking.” Only approved outpatient production recorded in SIA/SUS was included. Although the national protocol also allows CXL to be recorded as a hospital procedure, Hospital Information System (SIH/SUS) data were not included in this analysis. Accordingly, the study describes approved outpatient CXL production recorded in SIA/SUS rather than all CXL performed under SUS financing. Procedures performed exclusively in the private sector were also not captured.

Variables and descriptive indicators

The primary descriptive indicator was the annual number of approved outpatient procedures. Additional indicators included the number of federative units with at least one recorded procedure in a given year; the percentage share of each federative unit and macroregion; the combined share of the three highest-volume federative units; geographic concentration across federative units measured by the HHI; the effective number of federative units; the Gini coefficient; the first and last years of recorded production for each unit; the number of active years; and annual production according to provider legal category.

A federative unit was considered active in a given year if it recorded at least one approved procedure. The HHI was calculated as the sum of squared state shares of national production, with higher values indicating greater concentration. The effective number of federative units was defined as the reciprocal of the HHI and represents the number of equally productive units that would generate the observed level of concentration. The Gini coefficient was calculated across all 27 federative units, including zero values; values approaching 1 indicate a more unequal distribution.

Data analysis

Analyses were descriptive and covered the complete national aggregated series available for the study period. Absolute counts and proportions were calculated directly from approved procedure quantities. Annual percentage change was calculated as ((Vt−Vt−1)/Vt−1) × 100. Cumulative growth was calculated relative to the selected baseline year. The 2025-to-2018 production ratio was calculated as V2025/V2018, and the compound annual growth rate (CAGR) from 2018 to 2025 was calculated as ((V2025/V2018)^(1/7)^−1) × 100. The year 2017 was treated as a partial implementation period because the operational effects of Ordinance No. 486 began in the reporting cycle following its publication [12]; accordingly, 2018 was used as the baseline for long-term growth estimates.

For geographic concentration analyses, each federative unit’s share was calculated as its approved procedure count divided by the national total for the corresponding year. The HHI was calculated as the sum of the squared federative-unit shares on a 0-1 scale, and the effective number of federative units was calculated as 1/HHI. The Gini coefficient was calculated across all 27 federative units from their approved procedure counts, including units with zero recorded procedures. Zero-production federative units were retained as true zero values rather than treated as missing observations. No missing annual values were imputed.

Hypothesis tests and P values were not used because the data were not a probability sample; instead, they represented the full set of available aggregated records for the procedure. Analyses were performed in Python 3 (Python Software Foundation, Wilmington, DE, USA) and independently checked in a spreadsheet. Visualizations included a time-series plot, a federative-unit heatmap, a Pareto chart, and provider-category composition over time.

Ethical considerations

The study used exclusively publicly accessible, aggregated administrative data with no possibility of individual identification. Article 26, items II and V, of Brazilian National Health Council Resolution No. 674/2022 provides for exemption from CEP/Conep review for research using publicly accessible information and research conducted exclusively with data already available in aggregated form without the possibility of individual identification [18]. Accordingly, this study did not require review by the CEP/Conep system. There was no participant contact and no access to medical records or personal identifiers.

## Results

National production and data consistency check

Annual totals derived from the two independent tabulations of the same SIA/SUS dataset-by federative unit and by provider legal category-matched exactly in every year, demonstrating internal consistency between the extracted tabulations. From 2017 through 2025, 12,216 approved outpatient procedures were recorded. Annual production increased from 79 procedures in 2017 to 3,415 in 2025. Using 2018 as the baseline for long-term growth estimates, volume rose from 263 to 3,415 procedures, a 12.98-fold increase and a compound annual growth rate of 44.2% (Table 1).

**Table 1:** Annual outpatient production and federative-unit concentration indicators, Brazil, 2017-2025. SP: São Paulo; HHI: Herfindahl-Hirschman Index; FU: federative unit. The effective number of federative units was calculated as the reciprocal of the HHI (1/HHI), with HHI expressed on a 0-1 scale.

Year	Procedures	Active FUs	São Paulo (%)	Top 3 FUs (%)	HHI	Effective FUs	Gini
2017	79	4	21.5	92.4	0.375	2.66	0.906
2018	263	5	60.5	96.6	0.434	2.31	0.917
2019	883	7	83.0	95.1	0.699	1.43	0.938
2020	630	11	77.8	89.5	0.614	1.63	0.911
2021	1,175	12	79.9	87.7	0.644	1.55	0.909
2022	1,405	11	71.3	85.7	0.524	1.91	0.895
2023	1,644	15	70.3	83.8	0.508	1.97	0.880
2024	2,722	15	75.6	86.7	0.580	1.72	0.891
2025	3,415	16	69.1	85.7	0.497	2.01	0.893

**Figure 1:**
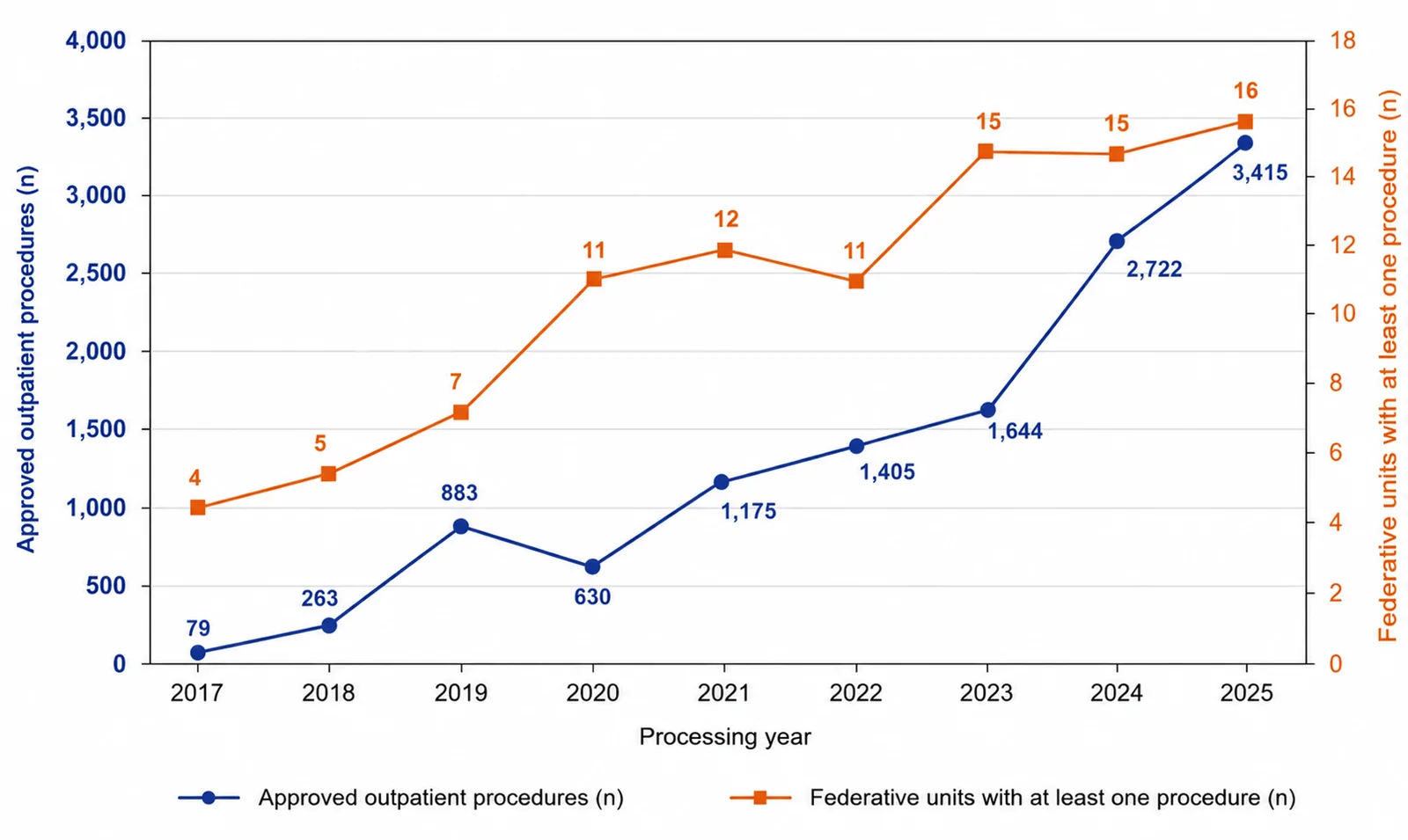
Approved outpatient corneal cross-linking production and number of federative units recording at least one procedure, Brazil, 2017-2025.

Production increased through 2019, declined by 28.7% in 2020, and resumed sustained growth from 2021 onward. Volume increased from 2,722 procedures in 2024 to 3,415 in 2025, a 25.5% rise and the highest annual total in the series. The 2020 decline coincided with the first year of the COVID-19 pandemic, although the study design does not support causal attribution (Figure 1).

Geographic distribution and concentration of recorded production

**Figure 2:**
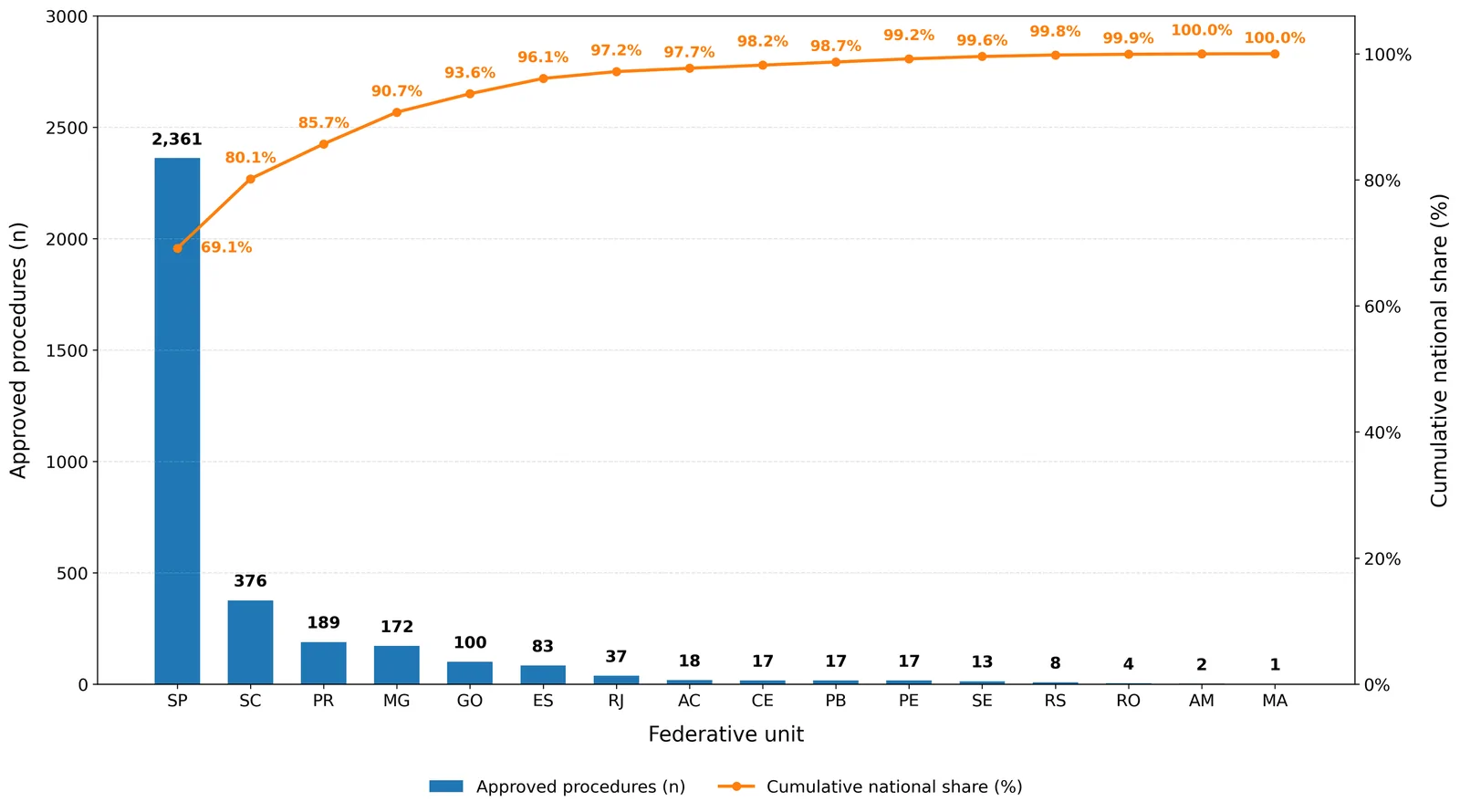
Pareto chart of approved outpatient corneal cross-linking production by federative unit, Brazil, 2025.

The number of federative units recording at least one procedure increased from five in 2018 to 16 in 2025. Concentration nevertheless remained high throughout the study period. In 2025, São Paulo recorded 2,361 procedures, representing 69.1% of the national total, while the three highest-volume federative units together accounted for 85.7% (Figure 2).

**Figure 3:**
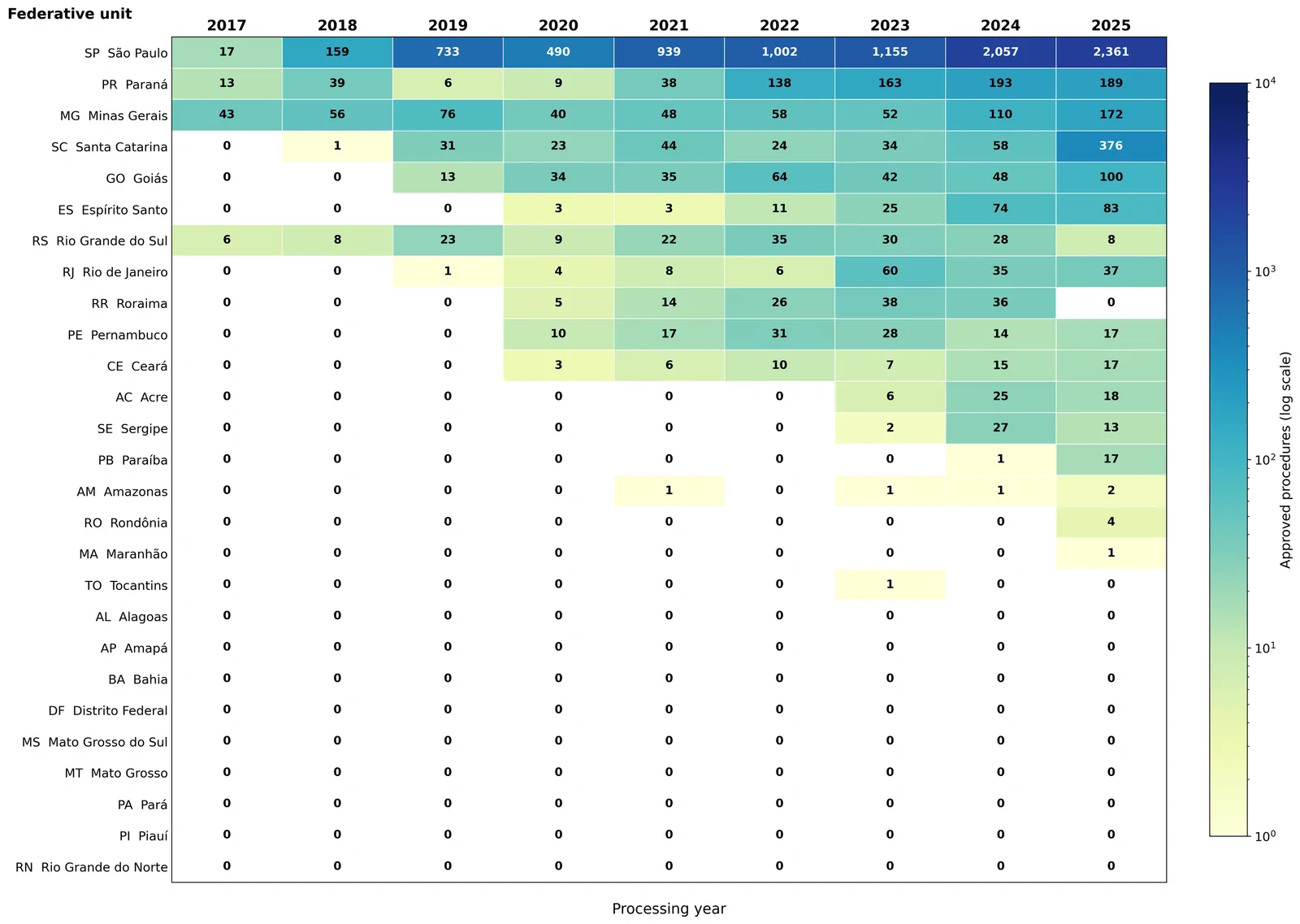
Heatmap of approved outpatient production by federative unit and year, ordered by cumulative procedure volume, Brazil, 2017-2025.

The HHI across federative units was 0.497 in 2025, corresponding to an effective number of 2.01 units. In other words, the distribution observed across 16 active units was as concentrated as a hypothetical distribution in which approximately two equally productive units accounted for the entire national volume. The Gini coefficient was 0.893. Thus, the growth in the number of participating units reflected nominal diffusion without a proportional redistribution of procedure volume (Figure 3).

Regional distribution

In 2025, approved outpatient CXL production recorded in SIA/SUS according to place of care was concentrated in the Southeast, which accounted for 2,653 procedures (77.7%), followed by the South with 573 (16.8%). The Central-West, Northeast, and North accounted for 2.9%, 1.9%, and 0.7% of recorded national outpatient production, respectively. The entire North recorded 24 approved outpatient procedures in 2025, compared with 2,361 recorded in São Paulo alone (Table 2). These figures refer to the location of the reporting establishment rather than patient residence and therefore should not be interpreted directly as regional differences in patient access or utilization.

**Table 2:** Cumulative approved outpatient production, 2017-2025, and approved outpatient production in 2025 by Brazilian macroregion.

Region	2017-2025, n	Cumulative share (%)	2025, n	2025 share (%)
North	178	1.5	24	0.7
Northeast	236	1.9	65	1.9
Southeast	9,918	81.2	2,653	77.7
South	1,548	12.7	573	16.8
Central-West	336	2.8	100	2.9

Federative-unit trajectories

Nine federative units had no approved outpatient CXL procedures recorded in SIA/SUS during the entire study period: Alagoas, Amapá, Bahia, the Federal District, Mato Grosso do Sul, Mato Grosso, Pará, Piauí, and Rio Grande do Norte. In 2025, 11 federative units had no approved outpatient CXL procedures recorded in SIA/SUS. Pará, the most populous state in the North, had no approved outpatient CXL production recorded in SIA/SUS during the study period. These findings do not establish the absence of CXL treatment in those federative units because patients may have traveled to another federative unit, procedures may have been recorded through SIH/SUS, care may have occurred exclusively in the private sector, or outpatient administrative records may have been incomplete.

State trajectories also showed substantial year-to-year variation. Santa Catarina increased from 58 procedures in 2024 to 376 in 2025; Goiás, from 48 to 100; and Minas Gerais, from 110 to 172. In contrast, Roraima decreased from 36 procedures to none, and Rio Grande do Sul from 28 to 8. Because the aggregated tabulations did not identify individual establishments, we could not determine whether these changes reflected the opening or closure of centers, changes in contracting, shifts in billing practices, or administrative reclassification.

Provider legal category

**Figure 4:**
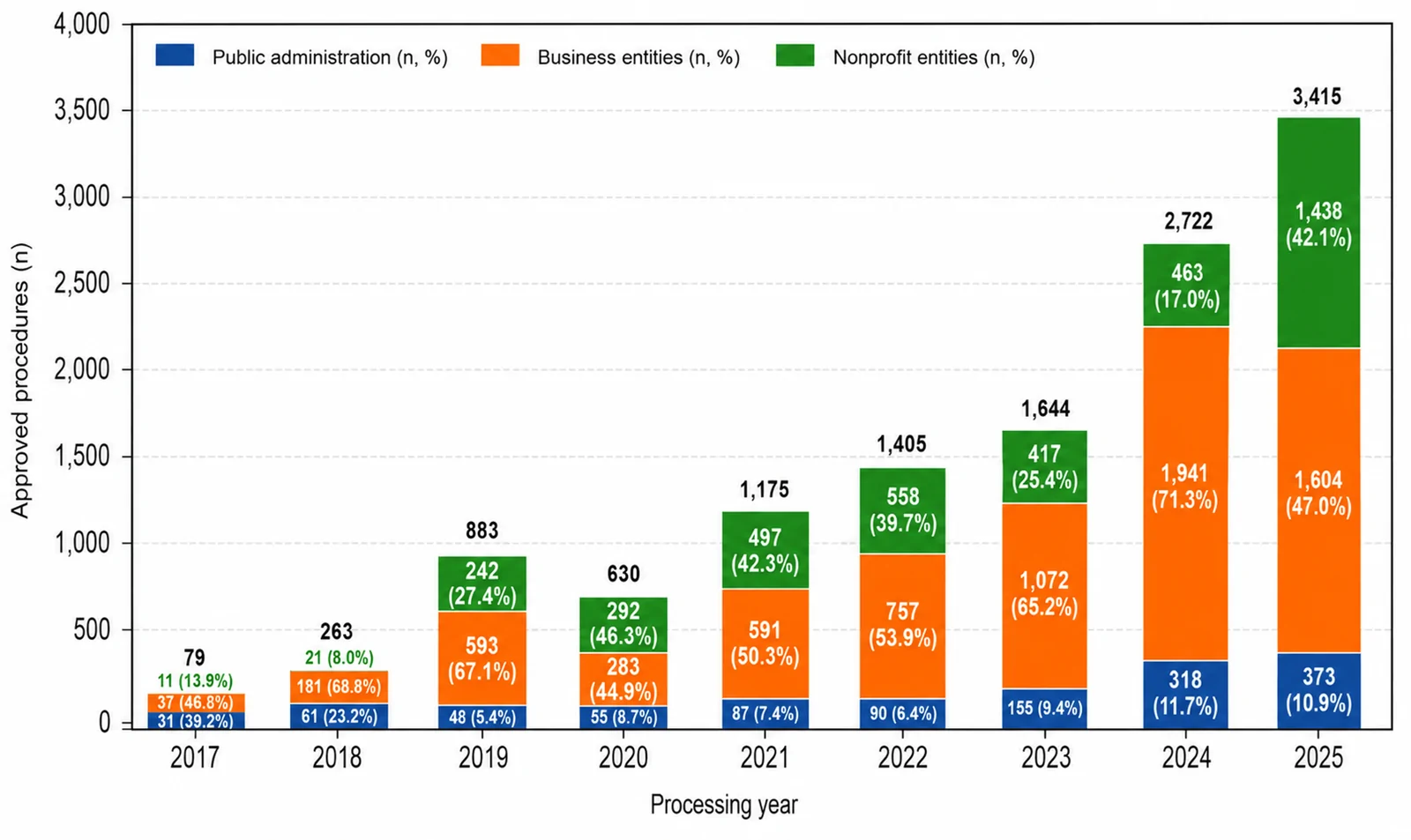
Approved outpatient corneal cross-linking production according to provider legal category, Brazil, 2017-2025.

In 2025, 373 procedures (10.9%) were recorded by establishments classified under public administration, 1,604 (47.0%) by business entities, and 1,438 (42.1%) by nonprofit entities. Overall, 89.1% of recorded production occurred in establishments outside public administration (Figure 4).

Between 2024 and 2025, production attributed to nonprofit entities increased from 463 to 1,438 procedures, whereas production attributed to business entities declined from 1,941 to 1,604 (Table 3). Because these categories are administrative, the data alone cannot establish a true replacement of providers. Part of the shift may reflect legal reclassification or changes in reporting practices.

**Table 3:** Approved outpatient production according to provider legal category, Brazil, 2017-2025.

Year	Public administration	Business entities	Nonprofit entities	Total
2017	31	37	11	79
2018	61	181	21	263
2019	48	593	242	883
2020	55	283	292	630
2021	87	591	497	1,175
2022	90	757	558	1,405
2023	155	1,072	417	1,644
2024	318	1,941	463	2,722
2025	373	1,604	1,438	3,415

The complete federative-unit profile, including cumulative production, first and last years of recorded activity, number of active years, and 2025 production, is presented in Table 4.

**Table 4:** Approved outpatient corneal cross-linking production and recorded activity profile by federative unit, Brazil, 2017-2025.

FU	Federative unit	Region	Total	First year	Last year	Active years	2025, n (%)
SP	São Paulo	Southeast	8,913	2017	2025	9	2,361 (69.1%)
PR	Paraná	South	788	2017	2025	9	189 (5.5%)
MG	Minas Gerais	Southeast	655	2017	2025	9	172 (5.0%)
SC	Santa Catarina	South	591	2018	2025	8	376 (11.0%)
GO	Goiás	Central-West	336	2019	2025	7	100 (2.9%)
ES	Espírito Santo	Southeast	199	2020	2025	6	83 (2.4%)
RS	Rio Grande do Sul	South	169	2017	2025	9	8 (0.2%)
RJ	Rio de Janeiro	Southeast	151	2019	2025	7	37 (1.1%)
RR	Roraima	North	119	2020	2024	5	0 (0.0%)
PE	Pernambuco	Northeast	117	2020	2025	6	17 (0.5%)
CE	Ceará	Northeast	58	2020	2025	6	17 (0.5%)
AC	Acre	North	49	2023	2025	3	18 (0.5%)
SE	Sergipe	Northeast	42	2023	2025	3	13 (0.4%)
PB	Paraíba	Northeast	18	2024	2025	2	17 (0.5%)
AM	Amazonas	North	5	2021	2025	4	2 (0.1%)
RO	Rondônia	North	4	2025	2025	1	4 (0.1%)
MA	Maranhão	Northeast	1	2025	2025	1	1 (0.0%)
TO	Tocantins	North	1	2023	2023	1	0 (0.0%)
AL	Alagoas	Northeast	0	-	-	0	0 (0.0%)
AP	Amapá	North	0	-	-	0	0 (0.0%)
BA	Bahia	Northeast	0	-	-	0	0 (0.0%)
DF	Federal District	Central-West	0	-	-	0	0 (0.0%)
MS	Mato Grosso do Sul	Central-West	0	-	-	0	0 (0.0%)
MT	Mato Grosso	Central-West	0	-	-	0	0 (0.0%)
PA	Pará	North	0	-	-	0	0 (0.0%)
PI	Piauí	Northeast	0	-	-	0	0 (0.0%)
RN	Rio Grande do Norte	Northeast	0	-	-	0	0 (0.0%)

## Discussion

This study identified a substantial increase in approved outpatient CXL production recorded in SIA/SUS from 2017 through 2025, accompanied by growth in the number of federative units with recorded activity. This geographic expansion of recorded production, however, did not result in a proportionate redistribution of procedure volume. In 2025, 16 federative units recorded activity, but the HHI corresponded to an effective number of only 2.01 units, with São Paulo accounting for 69.1% of recorded national outpatient production. The principal contribution of this analysis is therefore to demonstrate that broader geographic distribution of recorded production and reduced concentration of procedure volume are distinct dimensions.

These concentration metrics require careful interpretation. Both the HHI and Gini coefficient were calculated from absolute procedure counts across federative units with markedly different population sizes. They therefore quantify concentration of recorded production rather than inequality in access, coverage, or alignment with epidemiologic need. For context, São Paulo represented approximately 21.6% of Brazil's estimated population in 2025 but accounted for 69.1% of outpatient procedures. Although this comparison suggests concentration well beyond the state's population share, it does not substitute for rates calculated using the protocol-eligible population aged 15 to 45 years [12,19].

The clinical importance of national growth stems from the role of CXL in stabilizing progressive keratoconus. Randomized evidence and long-term studies demonstrate reduced progression in many treated patients [4-6]. Studies from Norway and the Netherlands reported fewer keratoplasties for keratoconus after CXL became available [7,8], and a Brazilian economic analysis found the procedure highly cost-effective within the SUS [9]. Realizing this population-level benefit, however, depends on timely diagnosis, documentation of progression, and practical availability within regional care networks.

The concentration observed in the North and Northeast warrants specific investigation. In 2025, the entire North recorded only 24 outpatient procedures, none of which occurred in Pará. These figures should not be interpreted as evidence of lower disease prevalence or the complete absence of care. Place of care files do not identify patient residence, and the national protocol permits hospital billing. Nevertheless, the low volume of approved outpatient production recorded locally in SIA/SUS identifies a limited administrative footprint of recorded outpatient production and raises hypotheses regarding interstate referral, diagnostic capacity, equipment availability, referral pathways, provider contracting, hospital-based billing, and completeness of outpatient administrative capture. These potential explanations cannot be distinguished using the present aggregated dataset and require further investigation.

The geographic concentration of recorded outpatient production observed in this study occurs within a broader context of documented regional differences in specialized health-service use and health-care infrastructure in Brazil [14,15]. In the field of health-technology implementation, federal guidance does not always specify the operational mechanisms needed to achieve consistent adoption across jurisdictions [13]. Although Ordinance No. 486 assigned local health authorities responsibility for organizing referral networks and services [12], the present series shows marked variation between federative units.

The 28.7% decline in 2020 coincided with the first year of the COVID-19 pandemic and was followed by sustained recovery. The current design cannot establish that the pandemic caused the decrease or separate its effect from administrative changes, installed capacity, or billing practices. Likewise, growth in 2024 and 2025 was not geographically uniform: São Paulo and Santa Catarina accounted for a substantial proportion of the recent increase.

Provider legal category adds an organizational dimension to the findings. In 2025, 89.1% of recorded production occurred in business or nonprofit entities. This does not imply that the procedures were delivered outside the SUS, which routinely contracts and partners with nonpublic providers. Rather, it indicates that outpatient production was predominantly located in establishments not classified under public administration. The sharp 2025 shift between business and nonprofit entities should be treated as a signal requiring investigation rather than definitive evidence of provider replacement.

This study did not measure access at the individual level. In other health systems, socioeconomic status, insurance coverage, and social vulnerability influence receipt of CXL [20]. Universal coverage through the SUS reduces direct financial barriers, but geographic distance, referral organization, access to corneal tomography, and service capacity may remain important determinants. Future studies should link municipality of residence, treating establishment, and travel flows to estimate geographic access more directly.

Implications for policy and research

National monitoring of CXL should extend beyond total procedure counts. Establishment-level continuity indicators, age-specific rates for the population aged 15 to 45 years, patient origin, travel distance, and geographic concentration would help distinguish a stable care network from sporadic administrative records.

Federative units with no or very low approved outpatient production recorded in SIA/SUS should be assessed individually. Potential explanations requiring investigation include absence of trained teams, limited access to corneal tomography, lack of equipment, absence of an established referral pathway, preferential use of hospital billing, interstate referral, or incomplete outpatient reporting. The present study cannot determine which, if any, of these mechanisms explains the observed pattern in a given federative unit, and each would require a different policy response.

The next analytical phase should incorporate SIH/SUS data, age-specific population estimates by federative unit, individualized SIA/SUS records, and the National Registry of Health Establishments (CNES). Linking these sources would permit the calculation of adjusted rates, the identification of persistently active centers, the estimation of interstate travel, and the evaluation of whether CXL expansion is associated with subsequent changes in keratoplasty for keratoconus.

The completeness and longitudinal stability of the provider legal category in the CNES should also be evaluated before changes in provider profile are interpreted. Concentration in a small number of institutions may increase procedural capacity but also create vulnerability to accreditation loss, contract interruption, or local service discontinuation.

Strengths and limitations

The study included the complete national outpatient series available over nine years, covered all 27 federative units, and cross-validated annual totals using two independent tabulations. Combining the number of active units with the HHI, effective number of federative units, and Gini coefficient demonstrated that broader territorial presence did not translate into a balanced distribution of production.

Several limitations should be considered. First, the approved quantity represents administrative procedure counts and cannot establish the number of unique patients, treated eyes, or distinct episodes of care. Accordingly, the 12,216 approved outpatient procedures recorded during the study period should not be interpreted as 12,216 individual patients. Bilateral treatment, repeat procedures, and administrative resubmissions cannot be disentangled in the aggregated dataset. Second, processing year may differ from the exact date of care. Third, place of care identifies the provider location rather than patient residence; the study therefore does not directly measure access, travel, or unmet need.

Fourth, age-specific population denominators were not used. Because federative units differ greatly in population size, the concentration indices reflect distribution of volume rather than equity in utilization. The contextual comparison between São Paulo's production and its share of the total population does not replace rates calculated for the protocol-eligible age group. Fifth, the study did not include SIH/SUS records despite the availability of hospital billing under the national protocol. Sixth, procedures performed exclusively in the private sector were not represented.

Seventh, provider legal categories are administrative and may change through reclassification. Individual establishments were not identified, preventing assessment of center-level persistence, capacity, quality, or continuity. Finally, recent administrative records may be revised; 2025 values should be rechecked immediately before final submission.

## Conclusions

Approved outpatient corneal cross-linking production recorded in SIA/SUS increased substantially from 2017 to 2025 and was recorded in a growing number of federative units. Procedure volume nevertheless remained highly concentrated: in 2025, São Paulo accounted for 69.1% of recorded outpatient procedures, the Southeast for 77.7%, and the national distribution was equivalent to approximately two equally productive federative units. Nine units had no approved outpatient CXL procedures recorded in SIA/SUS during the entire series. Most recorded production occurred in establishments classified outside public administration. These findings characterize the geographic distribution and concentration of recorded outpatient production rather than individual access, service availability, or population coverage. A fuller characterization of outpatient CXL production in the SUS will require rates adjusted for the eligible population, incorporation of SIH/SUS data, establishment-level identification, and analysis of flows between patient residence and place of care.
